# Benchmarking cell type annotation methods for 10x Xenium spatial transcriptomics data

**DOI:** 10.1186/s12859-025-06044-0

**Published:** 2025-01-20

**Authors:** Jinming Cheng, Xinyi Jin, Gordon K. Smyth, Yunshun Chen

**Affiliations:** 1 Bioinformatics Division, The Walter and Eliza Hall Institute of Medical Research, Parkville, VIC 3052 Australia; 2 Department of Medical Biology, The University of Melbourne, Parkville, VIC 3010 Australia; 3 School of Mathematics and Statistics, The University of Melbourne, Parkville, VIC 3010 Australia; 4 ACRF Cancer Biology and Stem Cells Division, The Walter and Eliza Hall Institute of Medical Research, Parkville, VIC 3052 Australia

**Keywords:** Spatial transcriptomics, Imaging-based, Cell type annotation, Reference-based annotation, Xenium, Single-cell

## Abstract

**Background:**

Imaging-based spatial transcriptomics technologies allow us to explore spatial gene expression profiles at the cellular level. Cell type annotation of imaging-based spatial data is challenging due to the small gene panel, but it is a crucial step for downstream analyses. Many good reference-based cell type annotation tools have been developed for single-cell RNA sequencing and sequencing-based spatial transcriptomics data. However, the performance of the reference-based cell type annotation tools on imaging-based spatial transcriptomics data has not been well studied yet.

**Results:**

We compared performance of five reference-based methods (*SingleR*, *Azimuth*, *RCTD*, *scPred* and *scmapCell*) with the marker-gene-based manual annotation method on an imaging-based Xenium data of human breast cancer. A practical workflow has been demonstrated for preparing a high-quality single-cell RNA reference, evaluating the accuracy, and estimating the running time for reference-based cell type annotation tools.

**Conclusions:**

*SingleR* was the best performing reference-based cell type annotation tool for the Xenium platform, being fast, accurate and easy to use, with results closely matching those of manual annotation.

## Background

Spatial transcriptomics technologies have been rapidly developed over the last several years [1–3]. Compared with single-cell RNA sequencing (scRNA-seq), spatial transcriptomics adds an additional spatial information layer. This makes it possible to investigate cell–cell communications and interactions, such as those between tumor cells and their microenvironment. Spatial transcriptomics platforms can be grouped into two broad categories: sequencing-based and imaging-based [4, 5]. Sequencing-based methods have the advantage of profiling whole transcriptome, but their resolution is usually at the spot or bin level, meaning each spot or bin may contain multiple cells. Popular sequencing-based spatial transcriptomics technologies include: 10x Visium [6], Slide-seq [7], GeoMx [8] and STOmics [9]. In contrast, imaging-based methods can achieve single-cell resolution but typically can only profile several hundred genes. The boundaries of individual cells are decided by cell segmentation algorithms [10–12]. Popular imaging-based spatial transcriptomics technologies include: 10x Xenium [6], MERSCOPE [13], CosMx [14], MERFISH [15] and STARmap [16]. Nowadays, imaging-based platforms have become increasingly popular as they enable the exploration of spatial gene expression profiles at the cellular level, despite their small gene panel size.

One key step before the downstream analysis is to annotate the cells of imaging-based spatial transcriptomics data correctly. However, the imaging-based spatial data harbors only several hundred genes, which sometimes makes it very hard and time-consuming to annotate cell types manually based on marker genes. Many reference-based cell type annotation methods have been developed for scRNA-seq data and shown very good performance [17], including *SingleR* [18], *Azimuth* [19], *scmap* [20], *scPred* [21], and *scMatch* [22]. Some reference-based cell type annotation methods have also been developed for sequencing-based spatial data, such as *RCTD* [23], *Spatial-ID* [24] and *Cell2location* [25].

Most of these software tools were developed in either the R or Python programming environments. In this study, we mainly focus on software tools implemented in R. One reason is the high level of similarity in functionality and underlying methodologies between these R and Python tools. For example, both *SingleR* and *scMatch* predict cell types based on the Pearson or Spearman correlation between the reference and the query datasets. Another reason is the wide user-base and ease of use of the Bioconductor and Seurat software tools due to their centralized version control and the high level of interoperability within their ecosystem [26, 27]. Hence, we utilized *SingleR*, *Azimuth*, *scmap*, *scPred* and *RCTD* for cell type annotation of imaging-based spatial transcriptomics data and benchmarked their performance. In addition, we compared the five methods with the manual annotation method based on marker genes.

Here, we used a public 10x Xenium data of Human HER2 + breast cancer for our benchmarking study. This is because Xenium is one of the most popular spatial technology platforms overall, due to 10x Genomics' established presence in the market and extensive user base. Additionally, this Xenium dataset includes profiles of two replicate samples along with a paired 10x Flex single-nucleus RNA sequencing (snRNA-seq) profile from one of the samples. Using a paired snRNA-seq profile as a reference is crucial for studying reference-based cell type annotation methods, as it minimizes the variability and inconsistencies between the reference and query datasets. In this study, we applied the marker gene-based manual annotation method and five reference-based cell type annotation methods (*SingleR*, *Azimuth*, *scmap*, *scPred* and *RCTD*) on the Xenium data to compare the performance of different reference-based methods and their similarity with the manual annotation method.

## Methods

### Data collection

Public Xenium and single-cell data of human HER2 + breast cancer from 10x Genomics [6] were downloaded from 10 × website: https://www.10xgenomics.com/products/xenium-in-situ/preview-dataset-human-breast. Xenium data of sample 1 (replicate 1) and sample 2 were used for this study. Paired 10x Flex single-nucleus RNA sequencing (snRNA-seq) data of sample 1 was downloaded and used as a reference for the reference-based methods. The provided cell type annotation files for both Xenium and snRNA-seq data were also obtained from the same website.

### Analysis of the snRNA-seq data

The *Seurat* (v4.3.0) [19] standard pipeline was performed on the snRNA-seq data of sample 1. For quality control, cells without 10x provided cell type annotation were removed. The filtered data was then normalized by the *NormalizeData* function. The top 1000 highly variable genes were selected by *FindVariableFeatures*, and normalized data of these genes were then scaled by *ScaleData* function. Principal component analysis (PCA) was performed to reduce dimensions by *RunPCA* function. Uniform manifold approximation and projection (UMAP) was performed by *RunUMAP* function on the first 30 principal components to further reduce the data into two dimensions. Potential doublets were predicted by *scDblFinder* [28]. Cell clusters were identified by *FindNeighbors* and *FindClusters* functions. *FindSubCluster* was used to identify sub clusters. To identify tumor cells, *inferCNV* analysis was performed to explore the copy number variations (CNVs) of chromosome segments, and it was done by comparing the expression of genes across positions of the snRNA-seq data with a normal reference scRNA-seq dataset. A public scRNA-seq sample of human normal breast tissue (N0233) [29] was used as the normal reference. The relative copy number expression value of each gene in each cell was obtained, and a value close to 0, 1, 2 indicates copy number loss, normal, copy number gain, respectively. Tumor cells were annotated based on copy number expression values from *inferCNV* analysis, and clusters with high CNVs were annotated as tumor. To evaluate whether a cell has higher CNVs than normal cells, we defined the *inferCNV* instability score for a cell to be the average squared deviation of normalized copy numbers from normality, $\text{S}=\frac{1}{n}{\sum }_{i=1}^{n}{\left({\text{V}}_{i}-1\right)}^{2}$, where *S* is the instability score, *V* is copy number expression value, *n* is the number of genes and *i* indexes genes. The instability score can then be used to compare CNVs between different clusters. Ductal carcinoma in situ (DCIS) was annotated based on 10x cell type annotation and marker genes. Clusters were annotated based on known marker genes.

### Analysis of the Xenium data

The *Seurat* standard pipeline was also performed on the Xenium data. There were some minor differences compared to snRNA-seq data analysis. For quality control, cells annotated as “Unlabeled” by 10x were removed, and other annotated cells from 10x were kept for analysis. The data was then normalized. Because Xenium data has only several hundred genes, the feature selection step was skipped, and all genes were used for data scaling. The dimension reduction (PCA and UMAP) and clustering steps are the same with snRNA-seq data analysis.

### Cell type annotation of Xenium data

For the manual annotation method, known marker genes based on our knowledge were used for annotating the cell type of each cluster. For the reference-based annotation methods, the snRNA-seq data of sample 1 was used as the reference, and potential doublets were removed to improve the accuracy of the reference data. Our manually annotated cell types for snRNA-seq data were set as the reference labels for predicting the cell types of Xenium data.

To prepare the reference for *Azimuth* method, *RunUMAP* was performed by setting “*return.model* = *TRUE*”, and *SCTransform* function in *Seurat* package was performed to re-normalize the snRNA-seq data, and *AzimuthReference* function in *Azimuth* (v0.4.6) package was used to generate the reference. To prepare the reference for *RCTD* method, *Reference* function in *spacexr* (v2.2.1) package [23] was used. *SingleCellExperiment* object format reference was prepared for *SingleR* and *scmap*, and *Seurat* object format reference was prepared for *scPred*.

Cell types were then predicted by each method using the prepared reference. For *SingleR* method, *SingleR* function in *SingleR* (v2.2.0) package was used for predicting cell types. For the *Azimuth* method, *RunAzimuth* function in *Azimuth* package was used for cell type annotation. For the *RCTD* method, *SpatialRNA* and *create.RCTD* functions in *spacexr* package were used to create the *RCTD* object, and *run.RCTD* function was used for cell type annotation. Many parameters in *run.RCTD* function were adjusted to keep all cells in the Xenium data: *UMI_min*, *counts_MIN*, *gene_cutoff*, *fc_cutoff*, *fc_cutoff_reg* were set to 0; *UMI_min_sigma* was set to 1; and *CELL_MIN_INSTANCE* was set to 10. For *scPred* method, *trainModel* function in *scPred* (v1.9.2) package was used to train a model for the reference, and *scPredict* function was used for cell type annotation. For *scmapCell* method, *indexCell* function in *scmap* (v1.22.3) package was used to create scmap-cell index, *scmapCell* function was used to project the index, and *scmapCell2Cluster* function was used to assign cell types. Default parameters were used in all the functions for all methods if not specifically mentioned above.

### Evaluation of performance of reference-based methods

To compare the performance of different reference-based methods, the composition of predicted cell types for each method was compared to that for the manual annotation method. We did not use the original cell annotation provided by 10x due to discrepancies between their annotation and the breast cancer literature. For example, the KRT15+ myoepithelial population, as originally annotated by 10x Genomics, should be labelled as luminal progenitor (LP) since they express ELF5 and MMP7 [30]. Additionally, certain subsets of some cell types are difficult to validate in the Xenium data, which makes the comparison more complicated. In our manual cell annotation, the three major normal epithelial cell populations were renamed as basal, LP and mature luminal (ML). The similarity of a reference-based method with the manual method was used to measure the performance of that method. To assess the accuracy of the predicted cell types, pseudobulk samples were constructed for each cell type in the spatial and reference snRNA-seq data. Spearman correlation between the two pseudobulk gene expression profiles of the same cell type was used to judge the accuracy of that cell type.

### Running time of reference-based methods

The most time-consuming steps of each method were recorded for making comparisons. The *SingleR*, *RunAzimuth*, *run.RCTD*, *scmapCell* functions are the most time-consuming steps for the *SingleR*, *Azimuth*, *RCTD* and *scmap* methods, respectively, whereas *trainModel* and *scPredict* are the most time-consuming steps for the *scPred* method. The running time for using all the cells in the reference snRNA-seq data was recorded for each method.

In addition, to explore the relationship of cell number of the reference data with running time, the reference snRNA-seq dataset of sample 1 was randomly sampled into smaller datasets containing 4000, 8000, 12,000, 16,000 and 20,000 cells, respectively. For the cell sampling process, 50 cells were first sampled into each cell type group to make sure there are sufficient cells in the new reference dataset, and the remaining cells were then randomly sampled and added into the new reference dataset. The running time was obtained for using each subsetted snRNA-seq dataset as reference for *SingleR*, *Azimuth* and *RCTD*.

## Results

### An overview of the reference-based methods

To identify the good cell type annotation methods for imaging-based spatial transcriptomics data, five referenced-based methods (*SingleR*, *Azimuth*, *RCTD*, *scPred* and *scmap*) developed for scRNA-seq data or sequencing-based spatial transcriptomics data were chosen for comparison (Table 1). *SingleR* is a popular and easy-to-use R package for cell type annotation of scRNA-seq data, and it is a method based on Spearman correlation. *Azimuth* is a popular R package developed by the *Seurat* team for transferring the labels of cells in a reference scRNA-seq data to a query dataset, and the *Seurat* referenced-based mapping pipeline is used for cell type annotation. *RCTD* is a referenced-based cell type annotation method implemented in *spacexr* package based on the robust cell type decomposition algorithm, and it was developed for sequencing-based spatial transcriptomics data, such as Visium. *scPred* is a machine learning based method to predict the cell types in a query dataset from a training scRNA-seq dataset, and the default model is support vector machine (SVM), which can be changed to other popular machine learning models. *scmapCell* is a method in *scmap* package and based on cosine distance kNN for cell type annotation of scRNA-seq data. *Azimuth* and *scPred* take a *Seurat* object as input, and *SingleR* and *scmapCell* take a *SingleCellExperiment* object as input. *RCTD* takes a *RCTD* object as input, and it can be easily constructed based on a *Seurat* object. *Seurat* and *SingleCellExperiment* objects are the two most widely used data structures in single-cell data analysis.

**Table. 1:** An overview of the reference-based methods used for this study

Method	Language	Package	Version	InputObject	ComputationalApproach
SingleR	R	SingleR	2.2.0	SingleCellExperiment	Spearman correlation
Azimuth	R	Azimuth	0.4.6	Seurat	Seurat reference-based mapping
RCTD	R	spacexr	2.2.1	RCTD	Robust cell type decomposition
scPred	R	scPred	1.9.2	Seurat	Support vector machine
scmapCell	R	scmap	1.22.3	SingleCellExperiment	Cosine distance based kNN

### An overview of the benchmarking workflow

**Fig. 1:**
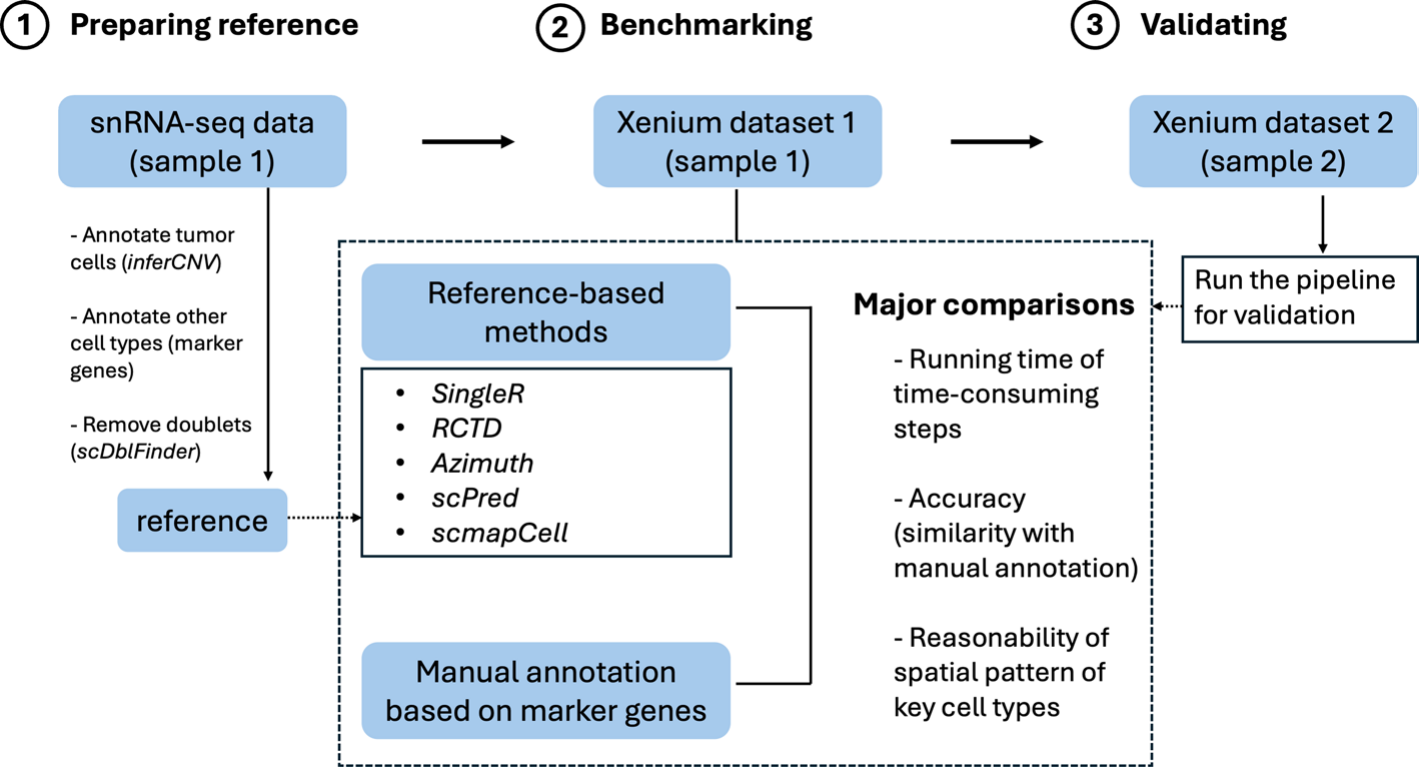
An overview of the benchmarking workflow. The benchmarking workflow includes (1) preparing a good single-cell RNA reference, (2) comparing reference-based methods using one Xenium dataset and (3) validating the results using another Xenium dataset

The workflow of benchmarking different reference-based cell type annotation methods on Xenium data has been shown (Fig. 1). The first step is to prepare a well annotated and accurate reference single-cell dataset. To make a good reference, we annotated tumor cells based on *inferCNV* analysis, annotated other cell types based on known marker genes and removed potential doublets. The next step is to predict cell types of a Xenium dataset using the reference for each reference-based method and using marker genes for the manual annotation method. We compared the running time of the time-consuming steps of different methods, the similarity between each reference-based method and the manual method (accuracy) and the spatial pattern of key cell types (e.g., Tumor). Finally, we performed the benchmarking workflow on another Xenium dataset to validate the results.

### Cell type annotation of the reference scRNA-seq data

**Fig. 2:**
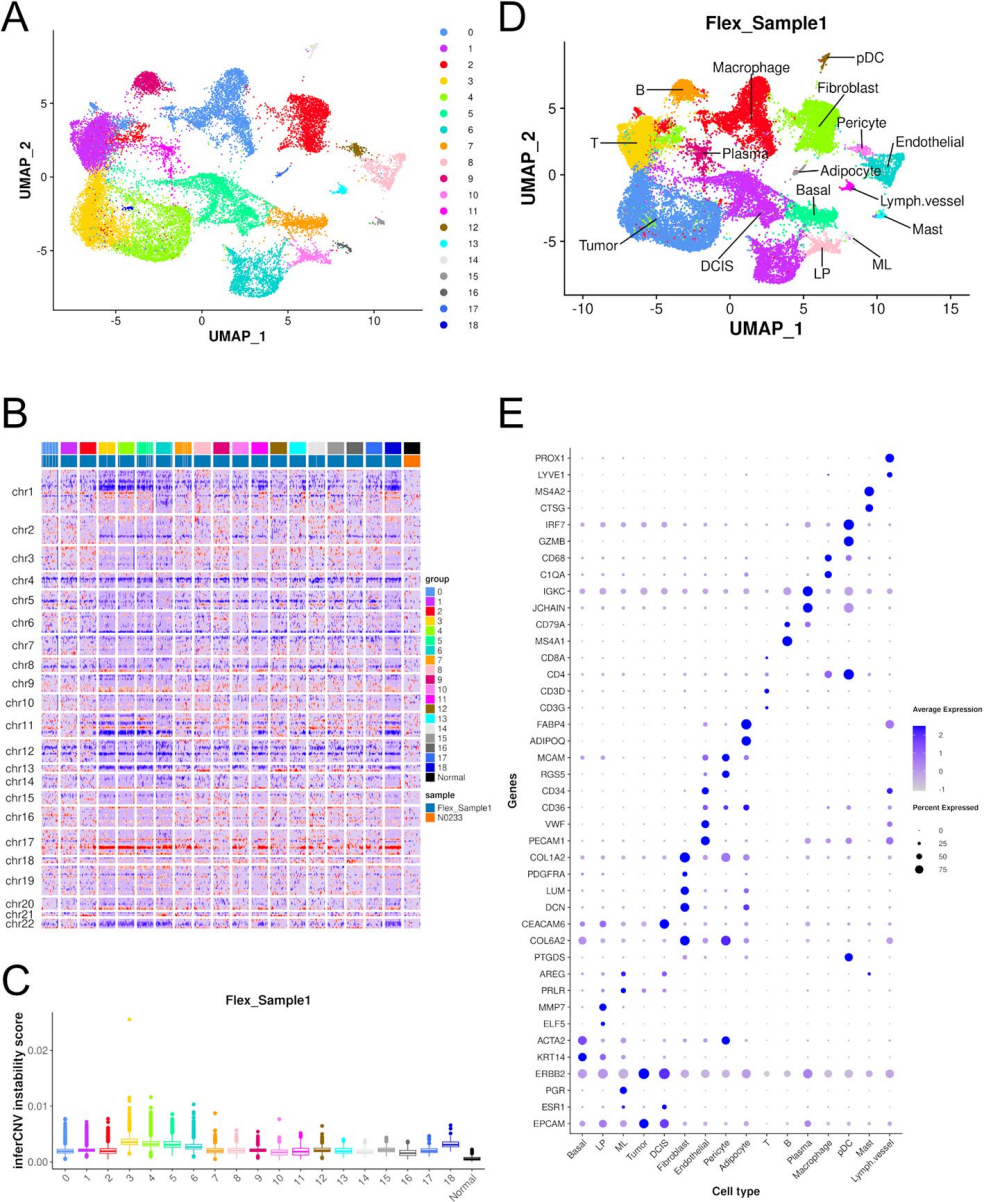
Cell type annotation of snRNA-seq data of sample 1. **A**. UMAP plot colored by clusters. **B**. Heatmap of CNVs from inferCNV. Clusters 3 to 6 show higher CNVs than other clusters. **C**. Boxplot of inferCNV instability scores. Clusters 3 and 4 (tumor) show a litter bit higher CNVs than 5 and 6 (DCIS). **D**. UMAP plot colored by cell types. **E**. Dot plot of marker genes used for cell type annotation

A good reference scRNA-seq dataset is important for the reference-based cell type annotation methods. The snRNA-seq technology can capture cell populations that are too fragile to be easily captured by scRNA-seq, such as adipocytes [31]. Therefore, it is preferable to use a snRNA-seq reference for imaging-based spatial transcriptomics data. The Flex snRNA-seq data of sample 1 from 10x Genomics was obtained and reanalyzed. Sample 1 is a human HER2 + breast cancer sample that harbors both tumor and DCIS cells according to the pathology report. Human breast tissue contains three major epithelial populations (basal, LP and ML) as well as the stromal and microenvironment cell populations. For quality control, 27,472 cells with 10x provided annotation were kept for analysis. *Seurat* pipeline was performed on the filtered snRNA-seq data, and 19 clusters were identified (Fig. 2A). Tumor cells were identified from *inferCNV* analysis (Fig. 2B, C). Both tumor (cluster 3 and 4) and DCIS (cluster 5 and 6) show high CNVs than other clusters. DCIS was distinguished from tumor by high expression of CEACMA6, a marker for DCIS. The cell clusters were manually annotated based on marker genes, and clusters assigned to the same cell type were merged (Fig. 2D). A dot plot of marker genes for each cell type was shown (Fig. 2E). *scDblFinder* was performed to identify the potential doublets, predicting 4280 cells as doublets. Doublets show expression pattern of two cell types and will affect accuracy of cell type prediction. Hence, the predicted doublets in the reference data were removed before cell type annotation for imaging-based spatial transcriptomics data. After removing the doublets, 23,192 cells in the snRNA-seq were kept for downstream analysis.

### Cell type annotation of Xenium data

Xenium data of sample 1 was obtained from 10x Genomics website and used as a representative of imaging-based spatial transcriptomics data. For quality control, only the cells with 10 × provided annotation were kept, and cells annotated as “Unlabeled” were further removed. This resulted in 159,226 cells retained for analysis. The *Seurat* pipeline was then performed on the filtered Xenium data. Clustering was conducted for the manual annotation method, while it was not required for the reference-based methods. Clusters were then manually annotated based on known marker genes. The pipeline of each reference-based method was performed to predict the cell type of each cell.

**Fig. 3:**
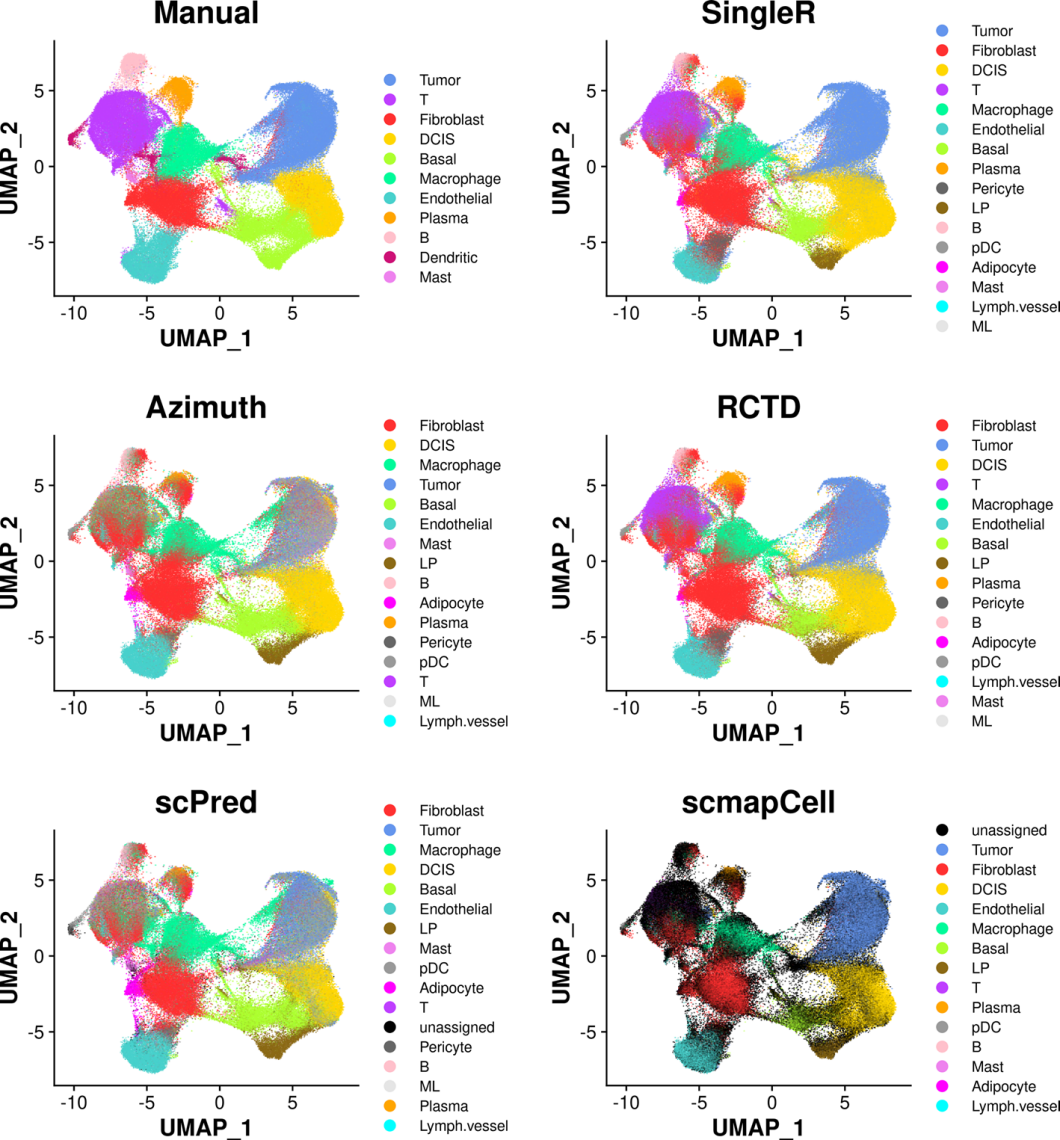
UMAP plots showing annotated cell types by different methods for Xenium data of sample 1

**Fig. 4:**
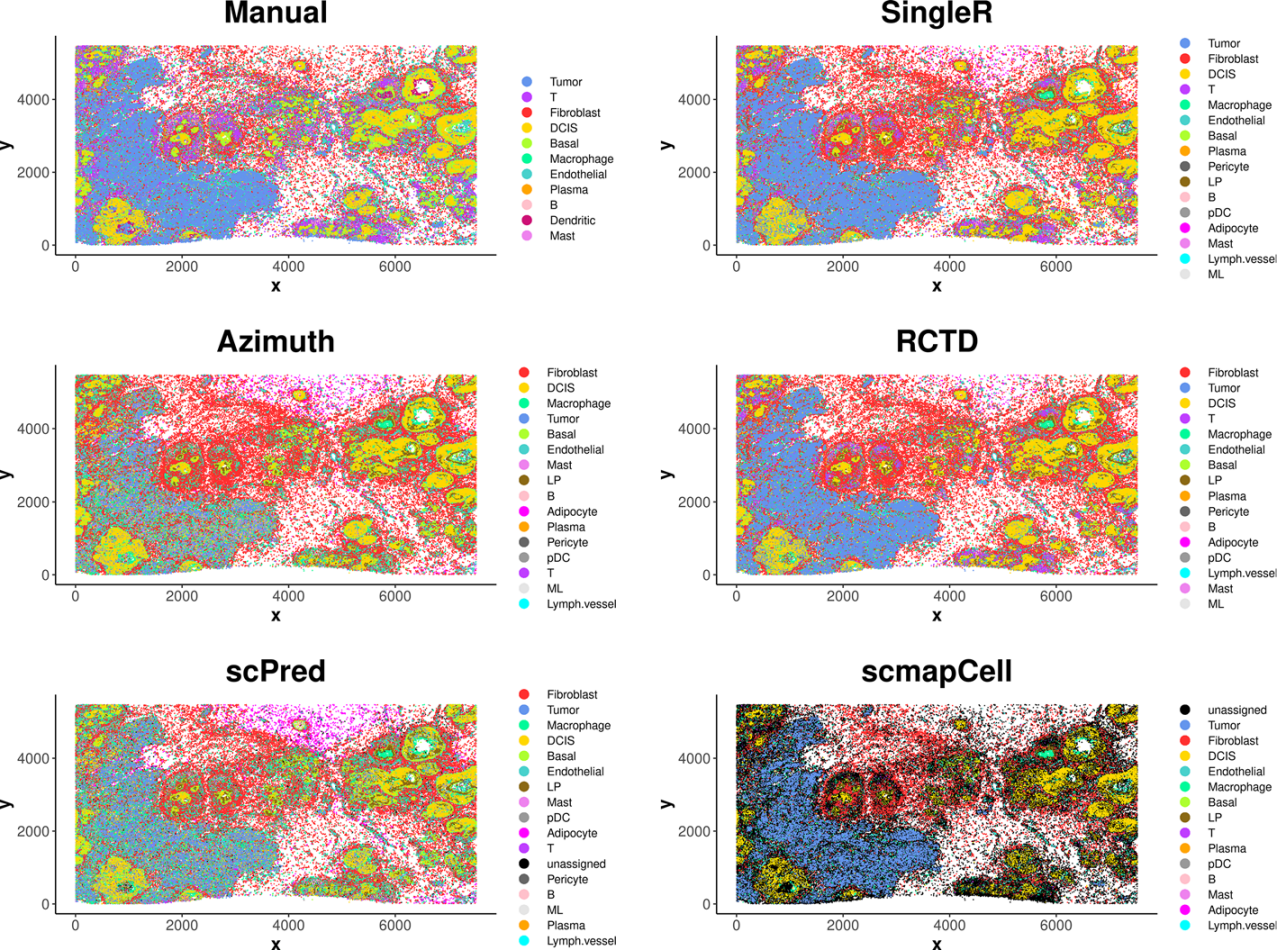
Spatial plots showing annotated cell types by different methods for Xenium data of sample 1

The annotated cell type by the manual method and five reference-based methods were shown in UMAP plots (Fig. 3) and spatial plots (Fig. 4). UMAP plots show the separated cell populations, and spatial plots show the spatial locations of different cell types. Because there are only 313 genes in the Xenium data of sample 1, it is hard to get good markers for all the cell types in the reference snRNA-seq from the gene panel. Hence, the number of manually annotated cell types is fewer than the cell types predicted by reference-based methods. On the other hand, the dendritic cells in the Xenium data could be annotated by the manual annotation method but could not be labelled by the reference-based methods due to difficulties distinguishing them in the reference snRNA-seq data. Hence, the manual annotation has the advantage of identifying cell types that are not observed in the reference snRNA-seq data. The basal population annotated by the manual method were further split into basal and LP populations by the reference-based methods (Fig. 3). The basal and LP cells were assigned to one cluster because of the similarity of the two cell types and low number of genes in the Xenium data, which makes them hard to be separated by clustering. The reference-based methods, on the other hand, perform annotation at the cell level and have the advantage of assigning each cell to the most possible cell type regardless of clustering.

The tumor and DCIS cells can be well visualized and separated in the spatial plots (Fig. 4). DCIS cells are inside the duct while tumor cells are spread outside of the duct, and they can be distinguished by the different patterns. Tumor cells could be well annotated by the manual method, *SingleR* and *RCTD*, while many of them were incorrectly annotated as other cell types by *Azimuth*, *scPred* and *scmapCell*. This suggests that the manual method, *SingleR* and *RCTD* have better performance in distinguishing tumor cell type from other cell types.

### Performance of different cell type annotation methods

**Fig. 5:**
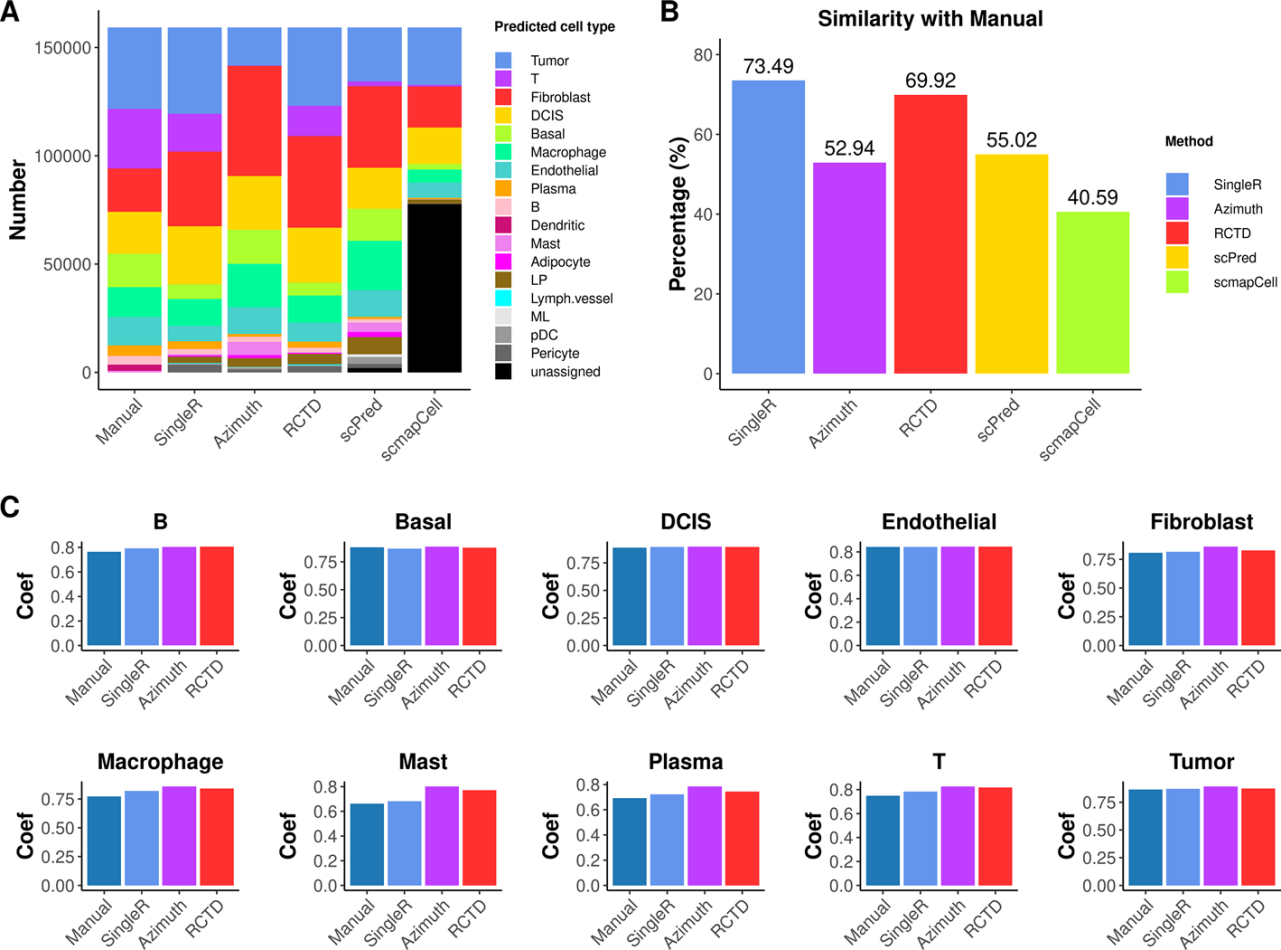
Performance of different cell type annotation methods for sample 1. **A**. Cell type composition for each method. **B**. Similarity of each reference-based method with manual annotation method. **C**. Spearman correlation coefficient of predicted cell type in spatial data with the same cell type in reference snRNA-seq data

The performance of the manual annotation method is good because the cell types were annotated carefully using known marker genes. Both the UMAP pot and spatial plot for the manual annotation method also show positive results. Although some cell types lacking marker genes could not be manually annotated, the cell type annotation of the majority cells are correct. The goal of reference-based cell type annotation is to minimize manual effort while ensuring that the annotation results closely match those obtained through manual cell type annotation. Hence, the manual annotation was used as the potential ground truth for comparing reference-based methods. The similarity of cell type composition of different reference-based methods with the manual annotation method was shown in the barplot (Fig. 5A). The proportion of tumor cells from *SingleR* and *RCTD* is quite similar to the manual method. Fewer tumor cells were annotated by *scPred* and *scmapCell*, and *Azimuth* gave fewest tumor cells. The proportion of DCIS cells is reasonable for all methods. T cells and macrophages are the two major immune cell populations in breast cancer microenvironment, and biologically reasonable proportions of T cells and macrophages were obtained from the manual method, *SingleR* and *RCTD*, while few T cells were annotated by *Azimuth*, *scPred* and *scmapCell*. Most cells were annotated as “unassigned” by *scmapCell*, and a small proportion of cells were annotated “unassigned” by *scPred*. When looking at the overall similarity with the manual method, *SingleR* and *RCTD* are the best methods, showing 73.49% and 69.92% cell type annotation consistency with the manual method (Fig. 5B). The similarity for *Azimuth*, *scPred* and *scmapCell* is below 60%. Hence, *SingleR* demonstrates the best performance, with *RCTD* also showing strong results. *Azimuth* is not so good, and *scPred* performs similarly with some cells remaining unassigned. *scmapCell* shows the worst performance and is not recommended.

To further explore the accuracy of annotated cell types by different methods, we constructed pseudobulk samples [32, 33] and compared the similarity of annotated cell types in the spatial data with that in the snRNA-seq data. *scPred* and *scmapCell* were not included for comparison because they are not of good performance. Spearman correlation coefficients between the spatial pseudobulk and snRNA-seq pseudobulk samples were calculated to assess the similarity for each cell type. The Spearman correlation coefficients are very high (> 0.7) for all the common cell types of four methods between spatial and single-cell data (Fig. 5C). This suggests that the manually annotated cell types and predicted cell types by the reference-based methods are of high accuracy.

### Running time of different methods

**Fig. 6:**
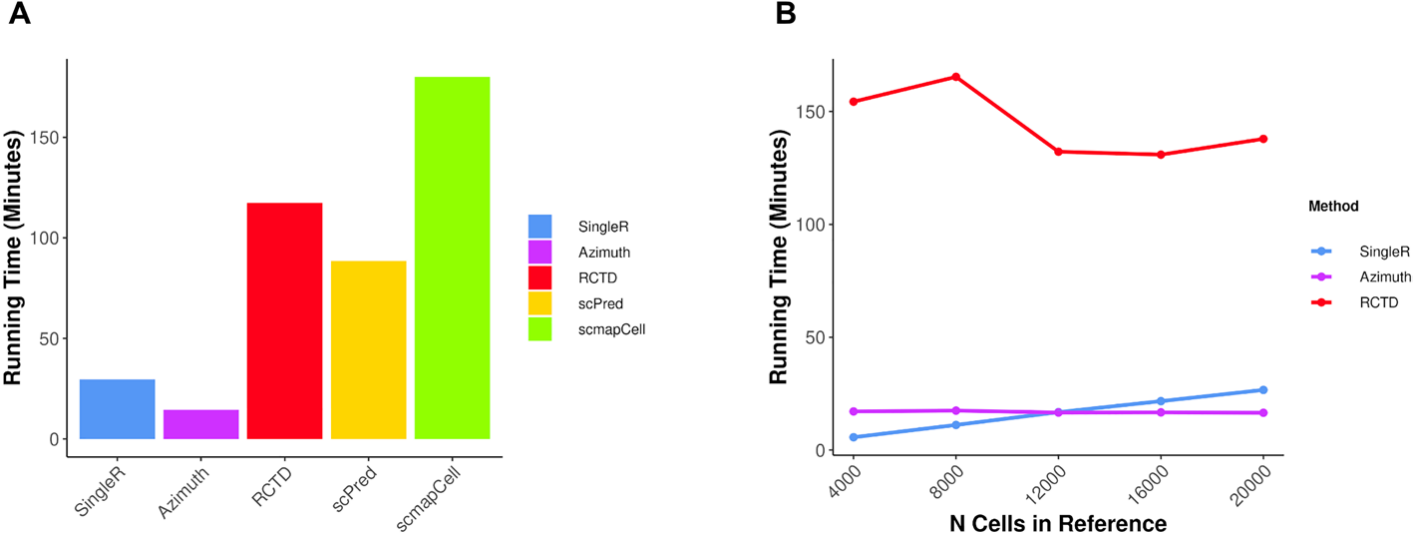
Running time of reference-based methods for sample 1. **A**. Running time for all reference-based methods using all cells in the reference. **B**. Running time for three reference-based methods using subsetted references

The manual annotation method usually takes 5–30 min but can take longer, and it requires good knowledge of markers for cell types in the biological data. The running time of the time-consuming steps of each reference-based method was recorded for using all the 23,192 cells in the reference snRNA-seq. *Azimuth* is the fastest method taking about 14.6 min, followed by *SingleR* taking about 29.6 min. *scPred*, *RCTD* and *scmapCell* are relatively slow, taking about 1.5 h, 2 h, and 3 h, respectively (Fig. 6A).

The snRNA-seq dataset we used for this study has many more cells than a typical 10x scRNA-seq sample, which usually contains 5000–10000 cells. Hence, it is valuable to know the running time for references with difference cell numbers. We generated 5 smaller reference snRNA-seq datasets by randomly sampling 4000, 8000, 12,000, 16,000 and 20,000 cells, respectively. The running time was obtained for *SingleR*, *Azimuth* and *RCTD* using each of the 5 snRNA-seq data subsets as the reference. The running time of *SingleR* is positively correlated with the number of cells in the reference, while *Azimuth* and *RCTD* are less affected by the number of cells in the reference (Fig. 6B). *SingleR* and *Azimuth* have almost equal running time of about 17 min when cell number of the reference is 12,000, with *SingleR* being faster for smaller references. *RCTD* is relatively slow, taking more than 2 h for all the reference subsets. Overall, *SingleR* is the fastest method when using a typical 10x scRNA-seq sample as reference.

### Validation of the performance of different cell type annotation methods

To validate and strengthen the conclusions made from analysis of Xenium data of sample 1, we further analyzed Xenium data of sample 2 using the same pipeline and the same snRNA-seq data as the reference. For quality control, only cells with 10x-provided annotations that were not labeled as “Not Plotted” were used for analysis. The *Seurat* pipeline was performed on 140,352 cells that were kept after quality control. The manual annotation and each reference-based method were then performed to obtain the cell types.

**Fig. 7:**
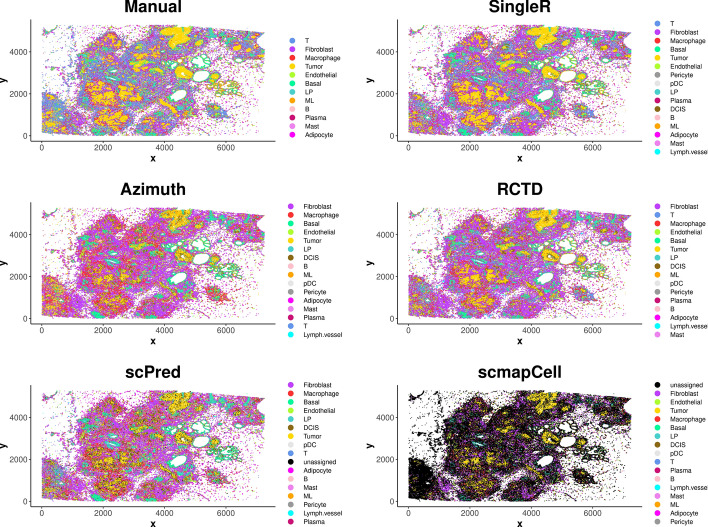
Spatial plots showing annotated cell types by different methods for Xenium data of sample 2

**Fig. 8:**
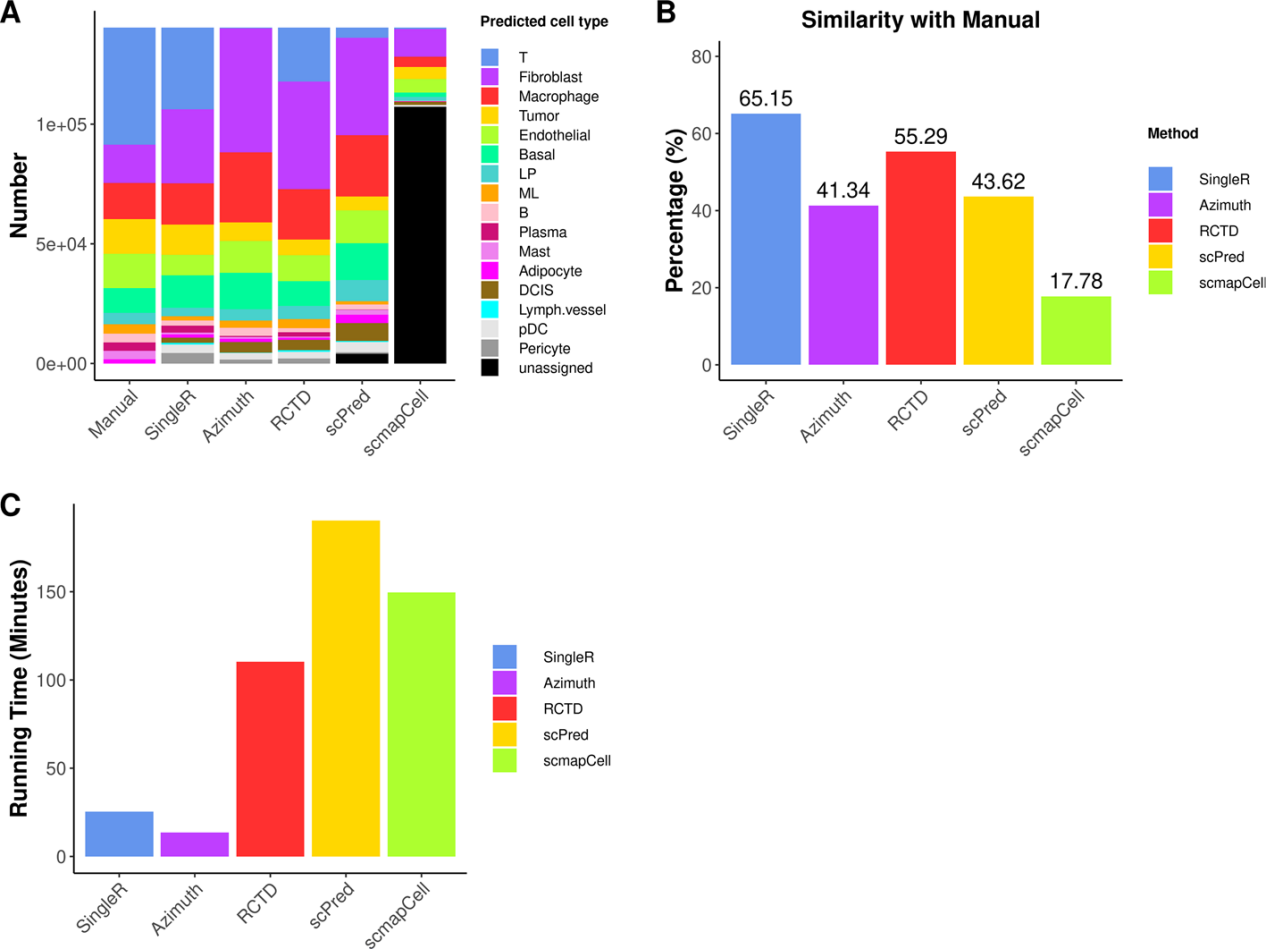
Performance and running time of reference-based methods for sample 2. **A**. Cell type composition for each method. **B**. Similarity of each reference-based method with manual annotation method. **C**. Running time for the reference-based methods using all cells in the reference

The spatial plots (Fig. 7) again show that *SingleR* and *RCTD* produce results similar to the manual method. In contrast, *Azimuth* and *scPred* are less similar to the manual method, while *scmapCell* shows significant discrepancies and performs poorly. The conclusions made from the spatial plots of sample 2 are consistent with that from sample 1. The barplot of cell type composition (Fig. 8A) shows that *scmapCell* has a high number of unassigned cells, while *scPred* also has some cells unassigned, which is undesirable. Additionally, *scPred* and *Azimuth* identify significantly fewer T cells than the manual method, *SingleR* and *RCTD*, which is consistent with the results from sample 1. Overall, *SingleR* and *RCTD* shows the highest (65%) and 2nd highest similarity (55%) to the manual method, making them the best two reference-based methods, while the other methods show lower similarity to the manual method (Fig. 8B). The running time for *Azimuth*, *SingleR*, *RCTD*, *scPred* and *scmapCell* is about 13 min, 25 min, 2 h, 3 h and 2.5 h, respectively (Fig. 8C). Altogether, the results from the analysis of sample 2 are similar to those from sample 1, reinforcing the conclusions made from the analysis of sample 1.

## Discussion

Imaging-based spatial transcriptomics has the power to investigate cell–cell interactions. Annotating the cell types accurately is important before the cell–cell interaction analysis and other downstream analyses. In this study, we applied one manual annotation method and five reference-based annotation methods on the Xenium data of human HER2 + breast cancer to find the best methods for cell type annotation of imaging-based spatial transcriptomics data. The manual annotation method is effective, but it requires good knowledge of biology and marker genes of different cell types. It also takes a substantial amount of manual effort and sometimes can be very time-consuming. The reference-based methods can save the manual work but require a well annotated reference scRNA-seq dataset. *SingleR* is the best reference-based cell type annotation method considering its easy-to-use workflow, good performance and fast computational speed. Hence, it is highly recommended when the cell number in the reference scRNA-seq data is not too big (e.g., 2000–8000). *RCTD* is a good method from the point of view of accuracy, though it is very slow and requires adjustment of many parameters. *Azimuth* performs poorly with this dataset but may be suitable for other datasets. *scPred* and *scmapCell* have poor performance and not recommended.

One limitation of this study is that only two samples from one of the imaging-based technologies (Xenium) were analyzed. We did not include other popular imaging-based spatial technology platforms, such as MERSCOPE and CosMx, due to the lack of public data resources containing both spatial and single-cell profiles generated from the same tissue samples. Different platforms may have different features, such as the size of the gene panel, sensitivity and specificity of molecule detection, that could affect the performance of cell type annotation tools. However, for platforms with similar characteristics, the performance of the cell type annotation methods included in this study is not expected to change significantly. In terms of tissue type, there appears to be no reason why any of the cell type annotation methods would be cell type specific. Another limitation is that none of the methods included in this study utilize spatial and image information for cell type annotation. Incorporating this extra spatial or image information could potentially enhance the performance of cell type annotation for spatial data.

## Conclusions

In summary, we compared different cell type annotation methods on Xenium data and found that *SingleR* is a fast, effective, and reliable reference-based method, showing high similarity with the manual annotation method. It is recommended over other reference-based methods for cell type annotation in Xenium data.

## Data Availability

No datasets were generated or analysed during the current study.

## Abbreviations

CNV: Copy number variation
DCIS: Ductal carcinoma in situ
LP: Luminal progenitor
ML: Mature luminal
PCA: Principal component analysis
scRNA-seq: Single-cell RNA sequencing
snRNA-seq: Single-nucleus RNA sequencing
SVM: Support vector machine
UMAP: Uniform manifold approximation and projection
